# Supplementary material for: High Diet Quality Is Linked to Low Risk of Abdominal Obesity among the Elderly Women in China
Source: Nutrients. 2022 Jun 24;14(13):2623. doi: 10.3390/nu14132623 (PMC9268347; doi:10.3390/nu14132623)
Supplement: Supplementary file 1 [file nutrients-14-02623-s001.zip › nutrients-1781483-supplementary.pdf]

Table S1. Endpoint characteristics of participants by gender in CHNS.

Characteristics	Men							Women						
	Q1 (*n* = 152)	Q2 (*n* = 171)	Q3 (*n* = 188)	Q4 (*n* = 179)	Q5 (*n* = 199)	Q6 (*n* = 193)	*p* [2]	Q1 (*n* = 136)	Q2 (*n* = 147)	Q3 (*n* = 137)	Q4 (*n* = 141)	Q5 (*n* = 156)	Q6 (*n* = 166)	*p* [2]
CDGI-E scores [1]	28.88	36.46	41.36	46.39	51.48	59.13	<0.001	31.22	39.53	45.63	50.40	55.26	63.74	<0.001
Age (y)[1]	71.10	71.81	73.31	72.91	72.83	71.45	0.276	73.99	73.11	74.31	72.72	73.86	73.06	0.787
PA (%)														
Light	44.74	47.37	48.94	56.42	55.28	55.96	<0.001	41.91	47.62	48.18	44.68	36.54	46.99	0.803
Moderate	29.61	28.65	29.26	30.17	29.65	33.68		40.44	33.33	34.31	38.30	45.51	39.16	
Heavy	25.66	23.98	21.81	13.41	15.08	10.36		17.65	19.05	17.52	17.02	17.95	13.86	
Educational level (%)														
Less than primary school	57.89	48.54	53.72	49.72	39.20	41.45	<0.001	67.65	66.67	61.31	64.54	53.85	57.83	0.009
Completion of primary school	21.05	23.98	22.34	22.91	28.64	19.69		15.44	14.29	18.25	15.60	21.15	16.27	
Middle school or advanced	21.05	27.49	23.94	27.37	32.16	38.86		16.91	19.05	20.44	19.86	25.00	25.90	
Geographic region (%)														
Central	50.00	50.29	41.49	32.96	33.17	35.75	0.110	45.59	36.73	40.15	34.75	26.92	22.29	0.003
East	18.42	16.37	22.87	31.28	33.17	35.75		26.47	27.89	20.44	20.57	29.49	38.55	
West	31.58	33.33	35.64	35.75	33.67	28.50		27.94	35.37	39.42	44.68	43.59	39.16	
Urbanicity index														
Low	44.08	40.94	31.91	31.28	21.11	19.17	<0.001	36.03	40.82	28.47	31.21	30.13	21.08	<0.001
Middle	30.92	38.60	32.98	30.17	30.65	25.39		41.18	33.33	38.69	36.88	25.64	25.30	
High	25.00	20.47	35.11	38.55	48.24	55.44		22.79	25.85	32.85	31.91	44.23	53.61	
Household income														
Low	32.89	38.60	32.98	24.02	28.64	28.50	<0.001	34.56	35.37	29.93	37.59	31.41	29.52	0.043
Middle	35.53	38.60	29.79	36.31	33.17	26.42		35.29	36.05	38.69	32.62	30.13	30.12	
High	31.58	22.81	37.23	39.66	38.19	45.08		30.15	28.57	31.39	29.79	38.46	40.36	
Ever smokers (%)	59.87	54.97	51.60	42.46	48.74	47.15	0.028	24.26	21.77	17.52	22.70	25.64	21.69	0.877
Energy intake (kcal/day) [1]	2027.66	1906.57	1889.79	1699.93	1706.07	1699.39	<0.001	1850.23	1722.46	1608.38	1643.42	1601.78	1589.78	<0.001
BMI (kg/cm^2)[1]	20.94	20.85	21.36	21.51	21.23	21.64	0.148	20.43	20.47	19.70	20.30	20.50	20.40	0.150
WC (cm)[1]	80.00	80.00	81.00	80.00	80.00	81.00	0.762	80.00	79.00	81.00	78.10	77.00	77.00	0.064
Abdominal obesity (%)	13.16	15.79	17.55	20.11	19.60	18.13	0.575	50.74	47.62	55.47	48.94	41.67	38.55	0.043
Overweight/general obesity (%)	11.92	11.38	17.65	14.94	14.72	13.68	0.589	7.46	12.58	10.53	10.22	10.32	17.47	0.127

Abbreviations: CDGI-E, China Elderly Dietary Guidelines Index; PA, physical activity; BMI, body mass index; WC, waist circumference; CHNS, China Health and Nutrition Survey. [1] Median. [2] Wilcoxon rank sum test for continuous and non-normally distributed variables, and chi-square test for categorical covariates.
